# In Vitro Effects of Fungal Phytotoxins on Cancer Cell Viability: First Insight into Structure Activity Relationship of a Potent Metabolite of *Cochliobolus australiensis* Radicinin

**DOI:** 10.3390/toxins14080517

**Published:** 2022-07-29

**Authors:** Veronique Mathieu, Stefano Superchi, Marco Masi, Patrizia Scafato, Alexander Kornienko, Antonio Evidente

**Affiliations:** 1 Department of Pharmacotherapy and Pharmaceutics, Université Libre de Bruxelles (ULB), Boulevard du Triomphe, Accès 2, 1050 Ixelles, Belgium; 2 ULB Cancer Research Center, Université Libre de Bruxelles (ULB), 1050 Bruxelles, Belgium; 3 Department of Sciences, University of Basilicata, Via dell’Ateneo Lucano 10, 85100 Potenza, Italy; stefano.superchi@uninbas.it; 4 Department of Chemical Sciences, University of Naples “Federico II”, Complesso Universitario Monte Sant’Angelo, Via Cintia 4, 80126 Napoli, Italy; marco.masi@unina.it (M.M.); evidente@unina.it (A.E.); 5 Department of Chemistry and Biochemistry, Texas State University, San Marcos, TX 78666, USA; a_k76@txstate.edu

**Keywords:** fungi, phytotoxins, radicinin, natural and hemisynthetic derivatives, anticancer, SAR study, *Cochliobolus australiensis*

## Abstract

Natural compounds have always represented an important source for new drugs. Although fungi represent one such viable source, to date, no fungal metabolite has been marketed as an anticancer drug. Based on our work with phytotoxins as potential chemical scaffolds and our recent findings involving three phytopathogenic fungi, i.e., *Cochliobolus australiensis*, *Kalmusia variispora* and *Hymenoscyphus fraxineus,* herein, we evaluate the in vitro anti-cancer activity of the metabolites of these fungi by MTT assays on three cancer cell models harboring various resistance levels to chemotherapeutic drugs. Radicinin, a phytotoxic dihydropyranopyran-4,5-dione produced by *Cochliobolus australiensis*, with great potential for the biocontrol of the invasive weed buffelgrass (*Cenchrus ciliaris*), showed significant anticancer activity in the micromolar range. Furthermore, a SAR study was carried out using radicinin, some natural analogues and hemisynthetic derivatives prepared by synthetic methods developed as part of work aimed at the potential application of these molecules as bioherbicides. This investigation opens new avenues for the design and synthesis of novel radicinin analogues as potential anticancer agents.

## 1. Introduction

Natural products and their hemisynthetic derivatives have always been a major source of new anticancer agents. Newman and Cragg [1] reported that from 1946 to 1980, more than 50% of anticancer drugs were natural products or their derivatives, while since 1981, the percentage of anticancer agents based on natural products or their derivatives has risen to 65%. Among them, many compounds are metabolites produced by bacteria, such as doxorubicin, daunomycin, mitomycin C, and bleomycin, all of which may be obtained from *Streptomyces*, as well as plants, like etoposide, teniposide, topotecan, paclitaxel, and the vinca alkaloids (vincristine, vinorelbine). Drugs based on fungal metabolites are also widely employed in medicine including penicillins, lovastatin (a cholesterol-lowering agent), echinocandin B (an antifungal), and cyclosporin A (an immunosuppressant) [2].

Numerous studies have highlighted fungal metabolites with promising anticancer activities, but to date, no fungus-derived agent has been approved as an anticancer drug [3,4,5]. We are particularly interested in ascomycetes, as these fungi are recognized as a significant source of compounds with phytotoxic activity [6]. Numerous phytopathogenic metabolites have been reported to exhibit potentially interesting biological activities which are important for human health [5,7,8,9,10,11]. For example, we showed that sphaeropsidin A, a phytotoxin isolated from the ascomycetes *Diplodia cupressi*, is able to induce rapid shrinkage of cancer cells. This was shown to be due to the targeting of the regulatory volume increase involved in cancer cell defense against cytotoxic agents [9]. Phytotoxic ophiobolins and particularly ophiobolin A, which is a sesterterpene characterized by a carbotricyclic ring shared with the phytotoxins fusicoccin and cotylenins, showed high potency against various cancer cell lines [10]. Ophiobolin A was isolated first as a phytotoxic agent for cereals produced by fungi belonging to different *Bipolaris* sp. and, more recently, as a potential bioherbicide from *Drechslera gigantea* [12].

**Figure 1:**
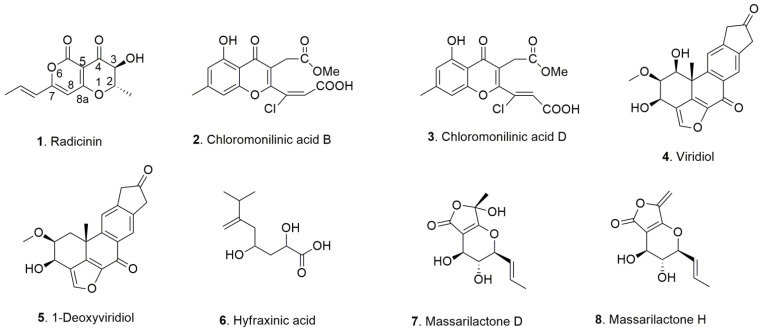
Structures of the fungal metabolites radicinin, chloromonilinic acid B, chloromonilinic acid D (**1**–**3**), viridiol, 1-deoxyviridiol, hyfraxinic acid (**4**–**6**), massarilactone D and H (**7**,**8**) that were produced by *Cochliobolus australiensis*, *Hymenoscyphus fraxineus* and *Kalmusia variispora*.

Following our results in recent studies aiming at isolating metabolites from other phytopathogenic fungi exerting interesting herbicidal and environmental effects, we decided to investigate their anticancer effects in vitro. Specifically, we studied some phytotoxic metabolites from *Cochliobolus australiensis*, *Kalmusia variispora* and *Hymenoscyphus fraxineus* (Table 1, Figure 1) [13,14,15,16].

As reported in Table 1, *C. australiensis* was proposed as a mycoherbicide to control buffelgrass (*Pennisetum ciliare* or *Cenchrus ciliaris*), which is a perennial grass that has become highly invasive in the Sonoran Desert of southern Arizona, causing severe damage by competition for water and nutrients with native plant species. The phytotoxic metabolites with potential herbicidal activity identified were radicinin (**1**), its 4,*O*-dihydroderivative radicinol, the 3-epimer of the latter and cochliotoxin, a new epoxy analogue of radicinin. When bioassayed on buffelgrass and non-host weeds, cochliotoxin, radicinin and 3-*epi*-radicinin showed strong phytotoxic activity while radicinol and 3-*epi*-radicinol were largely inactive [13]. In addition, from the organic extract of the same fungal culture filtrates, we isolated three chromanonacrylic acids, namely chloromonilinic acids B, C (**2**) and D (**3**). All three chloromonilinic acids were toxic against buffelgrass using two different bioassays [14]. *H. fraxineus* is the causal agent for ash (*Fraxinus excelsior* L.) dieback in Europe causing heavy losses to both forest heritage and wood industry. When grown in vitro, it produces viridiol, 1-deoxyviridiol and hyfraxinic acid (**4**–**6**), together with nodulisporiviridin M, and demethoxyviridiol. When tested on *Celtis australis* L., *Quercus suber* L., *Hedera elix* L., *Juglans regia* L. and *Fraxinus angustifolia* L. by leaf-puncture assay, only compounds **6**, **4** and dimethoxy-viridiol exhibited strong phytotoxic activity, while the other compounds were not toxic [15]. Two polyketides, identified as massarilactones D and H (**7** and **8**), were isolated from the organic extract of the culture filtrates of a strain of *K. variispora*, associated with grapevine trunk diseases in Iran. Both furanones showed phytotoxic activity on *Vitis vinifera* L. at variable concentrations and depending on the day of inoculation [16].

For our anticancer biossays, we selected fungal phytotoxins harboring different and original carbon chemical skeletons (Figure 1).

After our first screening assay, as radicinin (**1**) turned out to be the most potent compound, i.e., with growth inhibitory concentration in the micromolar range, we further conducted a first structure activity relationship (SAR) investigation by comparing the activity of **1** to that of some of its hemisynthetic and synthetic derivatives. The results of this study open research avenues for the chemical optimization of radicinin-based analogs as potential anti-cancer agents.

## 2. Results and Discussion

The fungal metabolites used were as follows: radicinin, chloromonilinic acids B and D (**1**–**3**), which were purified from the culture filtrates of *C. australiensis* [14]; viridiol, 1-deoxyviridiol, hyfraxinic acid (**4**–**6**), which were purified as mentioned above from the culture filtrates of *H. fraxineus* [15]; and massarilactones D (**5**) and H (**7** and **8**), which were purified from the culture filtrates of *K. variispora* [16]. Among them, radicinin (**1**) demonstrated promising bioherbicidal activity against the buffelgrass weed, showing high target-specific toxicity but low toxicity to native plants [17]. It also showed antifungal, insecticidal and plant growth regulatory activities [18], as well as antibiotic properties against Gram-positive bacteria, including *Staphylococcus aureus* and *Clostridium* sp. [19]. More recently, the ability of **1** to inhibit *Xylella fastidiosa*, a bacterium causing devastating diseases in many plants including grapevine and olive trees, has been demonstrated [20].

All the metabolites were identified by comparing their ^1^H NMR and ESIMS spectra with those reported in the previously cited literature. Their purity (>98%) was ascertained by ^1^H NMR and HPLC analyses.

All eight metabolites were assayed by the MTT method [21,22] to evaluate their anticancer activity against three human cancer cell lines, i.e., A549 non-small cell lung carcinoma (NSCLC), Hs683 oligodendroglioma and SKMEL-28 melanoma cells. The cell lines were chosen based on their relative resistance to apoptosis induction with the goal of identifying potential candidates that could overcome apoptosis-mediated drug chemoresistance. The Hs683 grade III oligodendroglioma cell line harbors 1p/19q co-deletion and is IDH mutated; as such, it is generally considered as chemo sensitive [23]. Although we observed better sensitivity of Hs683 to conventional cytotoxic agents in vitro in comparison to the U373 glioblastoma model [24], this might not be true for all agents. Dunn et al. [25] showed that Hs683 harbors only 5% of methylation in the MGMT gene promoter, a key gene for the repair of temozolomide-induced DNA damages [26]. The NSCLC A549 and the melanoma SKMEL-28 cell models have been shown to resist several pro-apoptotic insults. In particular, A549 is a heterogenous cell line with subclones that are highly resistant to chemotherapeutic insults [27]. In addition, its clinically relevant and well-known resistance to TRAIL-induced apoptosis [28] was previously shown to be overcome with natural products [29,30]. The melanoma SKMEL-28 model is BRAF mutated [31] and did not result in conventional apoptotic cell death under various plant metabolite treatments in vitro [31,32]. Cells were treated in our assay for 72 h at concentrations of 100 µM, 50 µM, 10 µM and 1 µM. Table 2 provides detailed IC_50_ values obtained for each cell line and compounds, while Table S1 in the Supplementary Information (SI) provides the residual viability of the cells at each concentration. Table S1 highlights the dose-dependent effects of the compounds. Among all compounds tested, radicinin (**1**) appeared to display the strongest anticancer activity (mean IC_50_ of 8.2 µM) followed by massarilactone H (**8**) (mean IC_50_ of 33.2 µM). Radicinin (**1**) was found to be as potent as cisplatin in vitro (mean IC_50_ of 8.4 µM Table 2).

**Figure 2:**
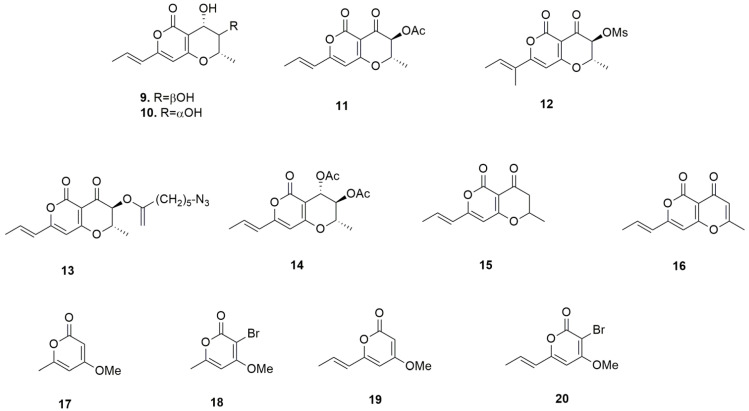
Structure of radicinol (**9**), 3-*epi*-radicinol (**10**), 3-*O*-acetyl radicinin (**11**), 3-*O*-mesyl radicinin (**12**), 3-*O*-(5-azidopentanoyl radicinin (**13**), 3,4-*O*,*O’*-diacetylradicinol (**14**), (±)-3-deoxyradicinin (**15**), 2,3-dehydroradicinin (**16**), 4-methoxy-6-methyl-2*H*-pyran-2-one (**17**), 3-bromo-4-methoxy-6-methyl-2*H*-pyran-2-one (**18**), (*E*)-4-methoxy-6-(propen-1-yl)-2*H*-pyran-2-one (**19**), and (*E*)-3-bromo-4-methoxy-6-(propen-1-yl)-2*H*-pyran-2-one (**20**), synthetic intermediates of (±)-3-deoxyradicinin (**15**).

Viridiol (**4**) and 1-deoxyviridiol (**5**) appeared to be poorly active with respective mean IC_50_ of 59.9 and 76.7 µM (Table 2). Hyfraxinic acid (**6**), massarilactone D (**7**) and chloromonilinic acids B and D (**2** and **3**) were inactive as anti-proliferative agents, with residual viability at 100 µM above 70% in comparison to the control condition (Table S1). Similarly, a recent study reported the absence of activity of chloromonolinic acids B, C and D on the U937 and K562 cell lines [33], confirming the lack of antiproliferative effects of these compounds toward cancer cells. Additionally, 9-*epi*-viridiol was previously shown to be poorly potent, with IC_50_ of 53.6 µM and 141 µM against HeLa and KB cells, respectively [34]. These results are comparable to those obtained herein with respect to its epimer, viridiol (**4**). Regarding the anti-cancer activity of massarilactones, our data suggest that the exomethylene group present in active massarilactone H (**8**) and not in inactive massarilactone D (**7**) may be an important structural feature. However, in a study by Teponno et al. [35], both massarilactones H and D were found to be inactive when tested at 1.3 mM against KB-3-1 cervix cancer cell line and L929 mouse fibroblasts. Of course, the difference in cancer type might explain this contrasting result. Thus, the most active compound was radicinin (**1**), whose IC_50_ of 8 µM, as observed here, is close to the mean GI_50_ of 11.4 µM found by the National Cancer Institute (NCI, Bethesda, USA) in their panel of 60 cancer cell lines, although in our assay, radicinin performed better against A549 and SKMEL-28 (GI_50_ of 20 and 16 µM, respectively, based on the results from the NCI; DTP datasearch, 6 June 2022). Among the few in vivo experiments conducted by the NCI with radicinin administered either intraperitoneally or subcutaneously, it appears that the acute approximate maximal tolerated dose in mice is 200 mg/kg. However, with repeated doses, this decreases, depending on the duration between administrations and the route of administration. For example, on the basis of daily intraperitoneal administration for 9 days, 150 mg/kg should be acceptable. This suggests that intraperitoneal administration actually leads to systemic exposure and cumulative toxicity. Interestingly, while radicinin and cisplatin exhibit similar potencies in terms of their IC_50_ against cancer cell lines (Table 1), the maximal tolerated dose of cisplatin in mice does not exceed 6 mg/kg after a single intraperitoneal administration [36]. The lower in vivo toxicity of radicinin might be due to its different pharmacodynamic and pharmacokinetic properties. These may be optimized through formulation, allowing highly toxic compounds be approved for marketing, as is, for example maytansin, that is now used in the clinic thanks to its conjugation to trastuzumab. It is worthy of note that radicinin was shown to have less pronounced effects on the viability of a non-cancerous cell line [37]. Nevertheless, no clear conclusion on benefits in tumor bearing mice could be reached after analyzing the experiments conducted by the National Cancer Institute. Furthermore, to the best of our knowledge, the anticancer mode of action of radicinin has never been studied. Considering that radicinin is mutagenic in AMES assays [38,39], particularly after nitrosylation, it might act as a DNA targeting agent. However, it is not teratogenic, sub-lethal or lethal on zebrafish (*Brachydanio rerio*) embryos up to 40 µM (five times higher than its IC_50_), which makes it a viable drug candidate [17]. In view of the chemical optimization that might be needed to improve the production and efficacy or to reduce toxicity, we conducted a preliminary SAR study using its natural and synthetic analogues **9**–**20**, as shown in Figure 2.

These compounds belong to the *α*-pyrone family, a well-known group of phytotoxins [40] which also display antifungal activity [41,42,43] and even anticancer activity [5,8]. In general, the presence of an α,β-unsaturated ketone group is already recognized as a factor which is important for biological activity, including anticancer properties, as this functionality could permit a Michael addition of a nucleophilic residue [44,45].

Radicinol (**9**) and its 3-epimer (**10**) were obtained from the culture filtrates of *C. australiensis*, while the 3-*O*-acetyl, 3-*O*-mesyl and 3-*O*-(5-azidopentanoyl) ester of radicinin (**11**–**13**), as well as the 3,4-*O*,*O*’-diacetyradicinol (**14**), were prepared by esterification of **1** and **9**, respectively. The synthetic analogues of radicinin (±)-3-deoxyradicinin (**15**) and 2,3-dehydroradicinin (**16**) were prepared according to a previously reported procedure [46], together with the four synthetic intermediates 4-methoxy-6-methyl-2*H*-pyran-2-one (**17**), 3-bromo-4-methoxy-6-methyl-2*H*-pyran-2-one (**18**), (*E*)-4-methoxy-6-(propen-1-yl)-2*H*-pyran-2-one (**19**), and (*E*)-3-bromo-4-methoxy-6-(propen-1-yl)-2*H*-pyran-2-one (**20**). The identity of all the compounds was ascertained by comparing their ^1^H NMR and ESIMS spectra with those reported in the literature [13,47,48]. Their purity (>98%) was ascertained by ^1^H NMR and HPLC. The obtained results are summarized in Table 3, with further details reported in Table S2 in SI. The results showed that radicinin (**1**) exerts the strongest in vitro anti-cancer activity, and that the esterification of the 3-hydroxy group did not substantially modify this activity. Under physiological conditions, the ester groups might be hydrolyzed, yielding the novel compound radicinin. Interestingly, among esters **11**–**13**, the potency decreases as the size of the ester group increases, possibly reflecting the difficulty associated with the hydrolytic removal of the acyl residue. The lack of activity in radicinol (**9**) is not in agreement with a previous study which showed that radicinol can induce apoptosis in a pancreatic cancer model and displays an IC_50_ between 10.5 to 25 µM, depending on the cell line studied [49]. However, our cell models were chosen for their resistance to apoptosis, and none of our cell lines was used in their study.

Considering the data obtained on the three cell lines, the lack of activity of radicinol (**9**), its 3-epimer (**10**) and the corresponding 3,4-*O*,*O’*-diacetyl derivative (**14**) showed that the carbonyl at C-4 is an important structural feature for anticancer activity, because, as anticipated, its presence allows a Michael addition of a nucleophile residue. The activities of (±)-3-deoxy- and 2,3-dehydro-radicinin (**15** and **16**) were slightly poorer than that of **1**, demonstrating that the 3-hydroxy group plays a minor role in the activity. Among the four synthetic intermediate methoxypyrones **17**–**20**, only **18** showed moderate anticancer activities, while **17**, **19** and **20** were completely inactive. Interestingly, 4-methoxy-2-pyrone is an important substructural unit of aurovertins B and D, i.e., fungal mycotoxins, showing strong antiproliferative activity against breast cancer cells but with little influence on normal cells [50]. As all four molecules are differently trisubstituted α-pyrones, their difference in activity might be due to the effect of the substituents on the Michael addition of a nucleophilic residue. The presence of the methoxy group at the β-position in all four compounds could reduce their reactivity due to steric hindrance, while the presence of bromine in α-position could instead increase this reactivity; see **18** vs. **17**. The difference between the moderate activity of **18** and the inactivity of **20** may be attributed to the presence of an ethenyl group at the δ-position in the latter, which could strongly reduce its reactivity in the Michael addition. Interestingly, the only active compound (**18**) does not bear a double bond conjugate to the pyrone which can act as a Michael acceptor. Therefore, it can be hypothesized that its cytotoxicity occurs through a different mechanism with respect to radicinin (**1**) and its derivatives. It is noteworthy that the results of the SAR study of **1** reported in Table 3 are in perfect agreement with those regarding phytotoxic activity against the host plant and other weeds using other natural analogues of **1** [47]. Importantly, we did not find strong variation in the responses among the three cell lines. Such results suggest that **1** might exert its in vitro anticancer activity through non-apoptotic pathways, making it an interesting candidate to develop further to combat chemoresistant cancers. Finally, as most molecules tested in Figure 1 and Figure 2 incorporate a lot of oxygen-containing functional groups and do not possess characteristic phenols or quinones, it is unlikely that this selection of molecules includes Pan-Assay Interference compounds (PAINs) [51].

## 3. Conclusions

This manuscript once again shows that phytotoxins could constitute potential anticancer agents in medicine [5,7]. Radicinin (**1**), isolated as the main phytotoxin from the weed pathogen *C. australiensis*, is the only compound showing strong anticancer activity among several other fungal metabolites (**2**–**8**) possessing different structural skeletons. Furthermore, its structural features are important for its anticancer activity, as we learned from a SAR study carried out using natural and synthetic analogues. Unfortunately, the low yield of **1** obtained from the fermentation of the fungus prevents its direct use in clinical trials. However, a more practical alternative to **1** is (±)-3-deoxyradicinin (**15**), which displays very similar cytotoxicity against the tested tumoral cell lines and may be obtained in a straightforward way through a novel synthetic strategy developed by some members of our team [46]. Therefore, **15** could be considered a promising new anticancer agent with potential for development. Our results warrant further studies to clarify the mode of action of these compounds in cancer cells.

## 4. Materials and Methods

### 4.1. Instruments, Chemical, Fungi and Plants

The optical rotations were recorded on a JASCO P-1010 (Tokyo, Japan) digital polarimeter; ^1^H NMR spectra were recorded at 400 MHz in CDCl_3_ on a Bruker (Karlshrue, Germany) spectrometer. Electrospray ionization mass spectra (ESI MS) were performed using the MS TOF system AGILENT 6230B (Milan, Italy). Analytical and preparative TLC were performed on silica gel plates (Merck, Kieselgel, Darmstadt, Germany) F_254_, 0.25 and 0.5 mm respectively; the spots were visualized by exposure to UV light and/or iodine vapors and/or by spraying first with 10% H_2_SO_4_ in methanol, and then with 5% phosphomolybdic acid in ethanol, followed by heating at 110 C for 10 min. Viridiol (**2**), 1-deoxyviridiol (**3**), hyfraxinic acid (**4**) and massarilactones D and H (**5** and **6**) were purified from the culture filtrates of fungi *Hymenoscyphus fraxineus* [15] and *Kalmusia variispora* [16] as previously reported. Radicinin (**1**), radicinol (**9**), its 3-epimer (**10**) and chloromonilinic acids B and C (**7** and **8**) were purified from the culture filtrates of *Cochliobolus australiensis* [13,14] as previously reported. 5-*O*-acetyl-, 5-*O*-mesyl and 5-*O*-azidopentanoyl ester of radicinin as well as diacetylradicinol (**11**-**14**) were prepared, respectively, from **1** and **9** according to the procedures previously reported [47]. Pyrones 4-methoxy-6-methyl-2*H*-pyran-2-one (**17**), 3-bromo-4-methoxy-6-methyl-2*H*-pyran-2-one (**18**), (*E*)-4-methoxy-6-(propen-1-yl)-2*H*-pyran-2-one (**19**), (*E*)-3-bromo-4-methoxy-6-(propen-1-yl)-2*H*-pyran-2-one (**20**), and dehydroradicinin (**9**) were intermediates obtained during the synthesis of (±)-3-deoxyradicinin (**15**) [46].

### 4.2. Cancer Cell Culture and Viability Assay

The three human cancer cell lines used in this study were A549 NSCLC (DSMZ code ACC107), Hs683 oligodendroglioma (ATCC code HTB-138) and SKMEL-28 melanoma (ATCC code HTB-72) cells. The cells were cultured in RPMI1640 (Invitrogen, Merelbeke, Belgium) media supplemented with 10% heat-inactivated fetal bovine serum (Greiner Bio one, Vilvoorde, Belgium), 4 mM glutamine and penicillin-streptomycin (200 U/mL and 200 μg/mL) (Invitrogen). Evaluation of the anti-proliferative effects of the compounds was conducted by using the colorimetric MTT (3-[4,5-dimethylthiazol-2-yl]) -2,5-diphenyltetrazoliumbromide; Sigma-aldrich, Merck, Hoeilaart, Belgium) assay, as previously described [52]. The assay is based on the capacity of viable cells to reduce the tetrazolium salt into formazan crystals. Briefly, 100 µL of cell suspensions was seeded in 96-well plates 24 h prior to the test at the following rates: 12,000/mL for Hs683, 13,000/mL for A549 and 18,000/mL for SKMEL-28. Then, the culture medium was refreshed with culture medium without or with each compound that was assayed in sextuplicate at concentrations of 100 µM, 50 µM, 10 µM and 1 µM for 72 h. At the end of the incubation period, cells were exposed to MTT solution in RPM1640 at 0.5 mg/mL for 3 to 5 h to allow living cells to reduce it into formazan. Plates were then centrifuged, and formazan crystals were dissolved in dimethylsulfoxyde. Absorbances were measured with a Biorad model 680XR (Biorad, Nazareth, Belgium) at a wavelength of 570 nm (with a reference of 610 nm). The absorbances of the control condition were set at 100% to normalize and compare the data. Mean residual viabilities of the treated cells compared to the untreated control condition and expressed in percentages are reported in Supplementary Tables S1 and S2, while the 50% growth inhibitory concentrations (IC_50_) are reported in the manuscript.

## Figures and Tables

**Table 1:** Metabolites isolated from the phytotopathogenic fungi *Cochliobolus australiensis*, *Hymenoscyphus fraxineus* and *Kalmusia variispora*.

Fungus	Host Plant	Disease	Metabolite	Ref
*Cochliobolus* *australiensis*	Buffelgrass (*Cenchrus ciliaris* L.)	Leaf Spots	Radicinin	[13]
			Radicinol	
			3-*epi*-Radicinin	
			Cochliotoxin	
			Chloromonilinic acid B	[14]
			Chloromonilinic acid C	
			Chloromonilinic acid D	
*Hymenoscyphus* *fraxineus*	Ash (*Fraxinus excelsior* L.)	Dieback	Viridiol	[15]
			1-Deoxyviridiol	
			Demethoxyviridiol	
			Nodulisporiviridin M	
			Hyfraxinic acid	
*Kalmusia variispora*	Grapevine (*Vitis vinifera* L.)	Trunk disease	Massarilactone D	[16]
			Massarilactone H	

**Table 2:** IC_50_ values of fungal phytotoxins from ascomycetes determined by means of the MTT colorimetric assay after 72 h of exposure. Data are expressed in µM as the mean ± SD of six replicates of one experiment.

Compound	A549	Hs683	SKMEL-28	Mean
cisplatin	6.3 ± 1.4	8.8 ± 0.8	10.0 ± 2.8	8.4
**1**. Radicinin	7.7 ± 0.6	8.7 ± 0.4	8.2 ± 0.2	8.2
**2**. Chloromonilinic acid B	>100	>100	>100	>100
**3**. Chloromonilinic acid D	>100	>100	>100	>100
**4**. Viridiol	65.5 ± 7.6	51.7 ± 5.3	62.4 ± 7.6	59.9
**5**. 1-deoxyviridiol	74.0 ± 6.6	64.9 ± 12.9	91.2 ± 18.8	76.7
**6**. Hyfraxinic acid	>100	>100	>100	>100
**7**. Massarilactone D	>100	>100	>100	>100
**8**. Massarilactone H	32.9 ± 3.5	31.6 ± 2.5	35.2 ± 2.8	33.2

**Table 3:** IC_50_ of radicinin and chemical analogs, determined by means of the MTT colorimetric assay after 72 h of exposure. Data are expressed in µM ± SD as the mean of six replicates of one experiment.

Compound	A549	Hs683	SKMEL-28	Mean
**1**. radicinin	7.7 ± 0.6	8.7 ± 0.4	8.2 ± 0.2	8.2
**9**. radicinol	>100	>100	>100	>100
**10**. 3-*epi*-radicinol	>100	>100	>100	>100
**11**. 3-*O*-acetylradicinin	7.0 ± 0.7	18.8 ± 2.5	7.9 ± 3.1	11.2
**12**. 3-*O*-mesylradicinin	8.5 ± 0.5	24.0 ± 0.2	6.9 ± 0.3	13.1
**13**. 3-*O*-(5-azidopentanoyl)radicinin	20.0 ± 2.1	23.9 ± 4.3	31.7 ± 1.5	25.2
**14**. 3,4*-O*,*O’-*diacetylradicinol	>100	>100	>100	>100
**15**. 3-deoxyradicinin	12.0 ±2.8	22.4 ± 2.0	20.4 ± 4.7	18.3
**16**. 2,3-dehydroradicinin	28.3 ± 1.6	30.5 ± 0.8	28.8 ± 2.1	29.2
**17**. 4-methoxy-6-methyl-2*H*-pyran-2-one	>100	>100	>100	>100
**18**. 3-bromo-4-methoxy-6-methyl-2*H*-pyran-2-one	59.7 ± 5.5	53.1 ± 2.8	73.2 ± 3.8	62.0
**19**. (*E*)-4-methoxy-6-(propen-1-yl)-2H-pyran-2-one	>100	>100	>100	>100
**20**. (*E*)-3-bromo-4-methoxy-6-(propen-1-yl)-2H-pyran-2-one	96.9 ± 8.6	99.4 ± 6.5	>100	>98.8

## Data Availability

Biological data are available upon request to Veronique Mathieu.

## Supplementary Materials

The following supporting information can be downloaded at: https://www.mdpi.com/article/10.3390/toxins14080517/s1, Table S1. Details on the residual viability observed at each concentration on each cell line. Table S2. Details the residual viability observed at each concentration on each cell line.
