# Supplementary material for: In Vitro Effects of Fungal Phytotoxins on Cancer Cell Viability: First Insight into Structure Activity Relationship of a Potent Metabolite of Cochliobolus australiensis Radicinin
Source: Toxins (Basel). 2022 Jul 29;14(8):517. doi: 10.3390/toxins14080517 (PMC9415302; doi:10.3390/toxins14080517)
Supplement: Supplementary file 1 [file toxins-14-00517-s001.zip › toxins-1789679-supplementary.pdf]

toxins

MDPI

Supplementary materials: *In vitro* effects of fungal phytotoxins on cancer cell viability: first insight into structure activity relationship of the potent metabolite of *Cochliobolus australiensis* radicinin.

Table S1. Details on the residual viability observed at each concentration on each cell line.

Compound	Concentration (μM)	% of residual viability A549	Hs683	SKMEL-28	mean
**1**, Radicinin	100	1	0	5	2
	50	1	0	7	2
	10	22	38	27	29
	1	72	71	83	75
**2**, Chloromonilinic acid B	100	72	78	76	75
	50	87	95	93	92
	10	99	108	105	104
	1	109	120	116	115
**3**, Chloromonilinic acid D	100	85	91	67	81
	50	112	119	88	106
	10	112	119	88	106
	1	136	146	107	130
**4**, Viridiol	100	11	9	9	10
	50	70	59	59	63
	10	125	106	107	113
	1	120	102	103	108
**5**, 1-deoxyviridiol	100	36	46	39	41
	50	63	82	70	72
	10	95	122	104	107
	1	104	135	114	118
**6**, Hyfraxinic acid	100	71	89	83	81
	50	87	109	102	99
	10	98	123	115	112
	1	103	129	121	118
**7**, Massarilactone D	100	55	64	60	59
	50	76	89	82	82
	10	104	123	114	114
	1	101	120	111	111
**8**, Massarilactone H	100	3	3	3	3
	50	6	5	5	5
	10	111	104	97	104
	1	117	110	102	110

Table S2. Details the residual viability observed at each concentration on each cell line. ND: not determined

Compound	Concentration (µM)	% of residual viability			mean
		A549	Hs683	SKMEL-28	
**1**, Radicinin	100	1	0	5	2
	50	1	0	7	2
	10	22	38	27	29
	1	72	71	83	75
**9**, Radicinol	100	67	53	97	72
	50	86	78	109	91
	10	87	78	103	89
	1	ND	ND	ND	ND
**10**, 3-*epi*-Radicinol	100	67	61	98	75
	50	89	79	105	91
	10	95	91	105	97
	1	85	86	100	90
**11**, 3-*O*-Acetylradicinin	100	3	2	5	3
	50	3	4	6	4
	10	33	63	47	48
	1	83	101	97	93
**12**-*O*-Mesylradicinin	100	3	1	3	2
	50	3	1	6	3
	10	40	76	24	47
	1	97	99	98	98
**13**, 3-*O*-(5-azidopentanoyl radicinin)	100	0	0	8	3
	50	3	2	10	5
	10	66	76	97	80
	1	ND	ND	ND	ND
**14**, 3,4-*O,O'*diacetyl radicinol	100	58	56	79	64
	50	80	77	90	82
	10	91	93	104	96
	1	ND	ND	ND	ND
**15**, 3-Deoxyradicinin	100	0	1	4	2
	50	0	1	5	2
	10	52	72	67	64
	1	93	91	110	98
**16**, 2,3-Dehydroradicinin	100	2	6	3	4
	50	9	11	4	8
	10	85	90	91	89
	1	93	97	102	97
**17**, 4-methoxy-6-methyl-2*H*-pyran-2-one	100	61	74	111	82

	50	83	103	119	102
	10	91	133	129	117
	1	90	106	132	109
**18**, 3-Bromo-4-methoxt-6-methyl-2*H*-pyran-2-one	100	7	17	15	13
	50	61	52	80	64
	10	108	85	113	102
	1	113	92	112	106
**19**, (*E*)-4-methoxy-6-(propen-1-yl)-2H-pyran-2-one	100	66	61	93	73
	50	96	83	105	95
	10	102	93	108	101
	1	101	91	107	99
**20**, (*E*)-3-bromo-4-methoxy-6-(propen-1-yl)-2H-pyran-2-one	100	48	49	61	53
	50	86	89	89	88
	10	110	111	106	109
	1	113	103	99	105
